# Perinatal optimisation for periviable birth and outcomes: a 4-year network analysis (2018–2021) across a change in national guidance

**DOI:** 10.3389/fped.2024.1365720

**Published:** 2024-04-17

**Authors:** J. Peterson, D. M. Smith, E. D. Johnstone, A. Mahaveer

**Affiliations:** ^1^ Faculty of Biology, Medicine and Health, The University of Manchester, Manchester, United Kingdom; ^2^ St Mary’s Maternity Hospital, Manchester University NHS Foundation Trust, Manchester, United Kingdom

**Keywords:** neonates, extreme preterm, periviable, perinatal, optimisation

## Abstract

**Introduction:**

The British Association of Perinatal Medicine (BAPM) released their revised framework for extremely preterm infant management in 2019. This revised framework promotes consideration of perinatal optimisation and survival-focused care from 22 weeks gestation onwards. This was a departure from the previous BAPM framework which recommended comfort care as the only recommended management for infants <23 + 0 weeks.

**Methods:**

Our study evaluates the clinical impact that this updated framework has had across the Northwest of England. We utilised anonymised network data from periviable infants delivered across the region to examine changes in perinatal optimisation practices and survival outcomes following the release of the latest BAPM framework.

**Results:**

Our data show that after the introduction of the updated framework there has been an increase in perinatal optimisation practices for periviable infants and an 80% increase in the number of infants born at 22 weeks receiving survival-focused care and admission to a neonatal unit.

**Discussion:**

There remain significant discrepancies in optimisation practices by gestational age, which may be contributing to the static survival rates that were observed in the lowest gestational ages.

## 1 Introduction

The periviable period refers to gestational ages where survival is possible, even if improbable. Where that limit lies depends on the timepoint, healthcare system and associated geographical location of the infant. In the United Kingdom in 2024, there is consideration for neonatal team presence and input for periviable births at or after 22 + 0 weeks (1, 2). Historically, intervention at periviability was considered futile. This was reflected in professional frameworks such as the British Association of Perinatal Medicine (BAPM) working group consensus issued in 2008 which cited comfort care as the only recommended management of infants <23 + 0 weeks (1).

With advances in neonatal research and medical technologies survival-focused care can be provided to infants from 22 + 0 weeks. There is an increasing body of evidence that active intervention at periviable gestation is associated with increased survival. The EXPRESS study from Sweden (2004–2007) reported 10% survival rate for 22 week infants (*n* = 51) and 53% for 23 week infants (*n* = 101) (3). For surviving infants, 40% of 22 week infants were reported to have mild disability and 49% of 23 week infants had no to mild neurodevelopmental impairment (30% had no and 19% had mild impairment) at 2.5 years corrected age (3). Subsequent data from the Neonatal Research Network of Japan (2008–2012) reported an increased survival rate at 46% for infants born at 22 + 0–22 + 6 weeks (*n* = 271), with 48% of surviving infants having no neurodevelopmental impairment at corrected age of 18 months (4). For infants born at 23 + 0–23 + 6 weeks, data from the Japanese research network also showed improved survival rates [mortality 27% (*n* = 686)] with no-mild neurodevelopmental impairment at 18 months corrected for 58% of survivors (4). Analysis of survival data for very preterm infants admitted to neonatal intensive care units (NICU) in England (2008–2014) was published in 2018 and demonstrated survival to discharge rates of 17.9% for 22 + 0–22 + 6 weeks (*n* = 12) and 35.9% for 23 + 0–23 + 6 weeks (*n* = 440) (5). In February 2019 Myrhaug et al., published their meta-analysis of survival and impairment of extremely premature infants which gathered and synthesised data from 65 studies conducted in high income countries (6). This meta-analysis found a mean survival rate of 24.1% for 22 + 0–22 + 6 week infants who had survived labour and been admitted to NICU. These data have established that intervention at periviable gestations cannot be considered futile.

In October 2019, BAPM launched its revised framework for perinatal management of the extreme preterm infant (1). This framework outlined an updated approach to this type of delivery encouraging a more holistic stance, rather than singular adherence to gestational age. The 2019 framework embraces the infant as an individual with its own set of protective attributes and risk factors and promotes clinicians using these factors, in deliberation with the parents, to determine whether active, survival-focused care or comfort care is most appropriate. The revised framework acknowledges the inherent inaccuracy in gestational age estimates which may be inaccurate by up to 5 days in either direction (1). This magnitude of discrepancy is significant at the limits of periviability where it can mean the difference between survival-focused care being offered or withheld. Whilst the BAPM Framework was certainly not solely for the periviable infant, the implications of the new BAPM framework for periviable infants were marked (7). With 1,000 liveborn infants delivered at <24 weeks every year across England and Wales the implications of the revised BAPM framework may have significant workforce and service level implications for neonatal networks (8). As outlined in the recent article by Smith et al., our region has proportionately high rates of implementing survival-focused care from 22 weeks gestation compared to the rest of England and Wales; with 52% of babies alive at the onset of labour receiving survival focused care in the North compared to other regions who report between 7% and 42% (9). Our study aimed to quantify perinatal optimisation approaches across the North West of England for these periviable infants including regional data regarding antenatal steroid and magnesium sulphate provision at 22 and 23 weeks, and to evaluate changes in optimisation rates across the time period the 2019 BAPM Framework came into effect. An additional aim was to assess the impact on the regional transport service. For the purposes of this article, the term *periviable* will be used in relation to infants born between 22 + 0 and 23 + 6 weeks. Whereas, *extremely preterm* will denote infants born between 22 + 0 and 24 + 6 weeks.

## 2 Methods

### 2.1 Data collection

Data were gathered through the North West Neonatal Operational Delivery Network [NWNODN (10)]. This ODN incorporates 22 neonatal units across the region, with two Special Care Units (SCBU; Level 1), twelve Local Neonatal Units (LNU; Level 2) and seven Neonatal Intensive Care Units (NICU; Level 3) with an additional surgical unit within a tertiary children's hospital (11). The study was reviewed and granted approval by the University of Manchester Psychology and Mental Health Divisional Review panel (2023-17791-31615). All data collected were anonymised. Data analysts from the NW ODN were able to collate data from the electronic patient record Badger.Net for all periviable deliveries (22 + 0–24 + 6 weeks) across the NW network. Data fields recorded were gestational age, birth weight, gender, ethnicity, level of birth location (NICU, SCBU, LNU), antenatal steroid provision [full (2× doses), incomplete (1× dose), none, not recorded], magnesium sulphate provision, delivery modality, admission temperature, neonatal outcome (survived/died) and complications [bronchopulmonary dysplasia (BPD), intraventricular haemorrhage (IVH) and retinopathy of prematurity (ROP)]. The Badger.Net definition for BPD was used, which is requirement for respiratory support at 36 weeks corrected/at time of discharge (12). Given the publication of the BAPM framework in late October 2019 we defined pre-BAPM as 1st January 2018–31st December 2019 and post-BAPM as 1st January 2020–31st December 2021.

**Inclusion criteria**

• Gestational age at birth 22 + 0–24 + 6 weeks (singletons and multiples were included)
• Episode one on Badger.Net occurring within the North West Network hospitals
• Alive at admission to the neonatal unit

**Exclusion criteria**

• Gestational age at birth ≤21 + 6 weeks or ≥25 + 0 weeks
• Infants born outside the North West Network. This exclusion was applied to infants repatriated back into the North West Network
• Intrapartum death or death in the delivery room

### 2.2 Neonatal transport service data

The North West has a dedicated neonatal transport service: Connect North West (13). Data were gathered from the ConnectNW cot bureau transfer database for all extremely preterm transfers (22 + 0–24 + 6 weeks) from inside the North West network between 1st January 2018 and 31st December 2021. This dataset included both in-utero and ex-utero transfers. Extracted data were anonymised.

### 2.3 Data analysis

Descriptive statistics, such as percentages, means and range values, were stratified by gestational age in weeks. Epoch comparisons (pre- vs. post-BAPM) were performed using *χ*^2^ (or Fisher's) test. Odds ratios were calculated for key variables and outcomes. Statistical significance was set at *p* < 0.05. The data were analysed using Excel (Version 16.69) and SPSS (Version 29.0) software.

## 3 Results

**Figure 1:**
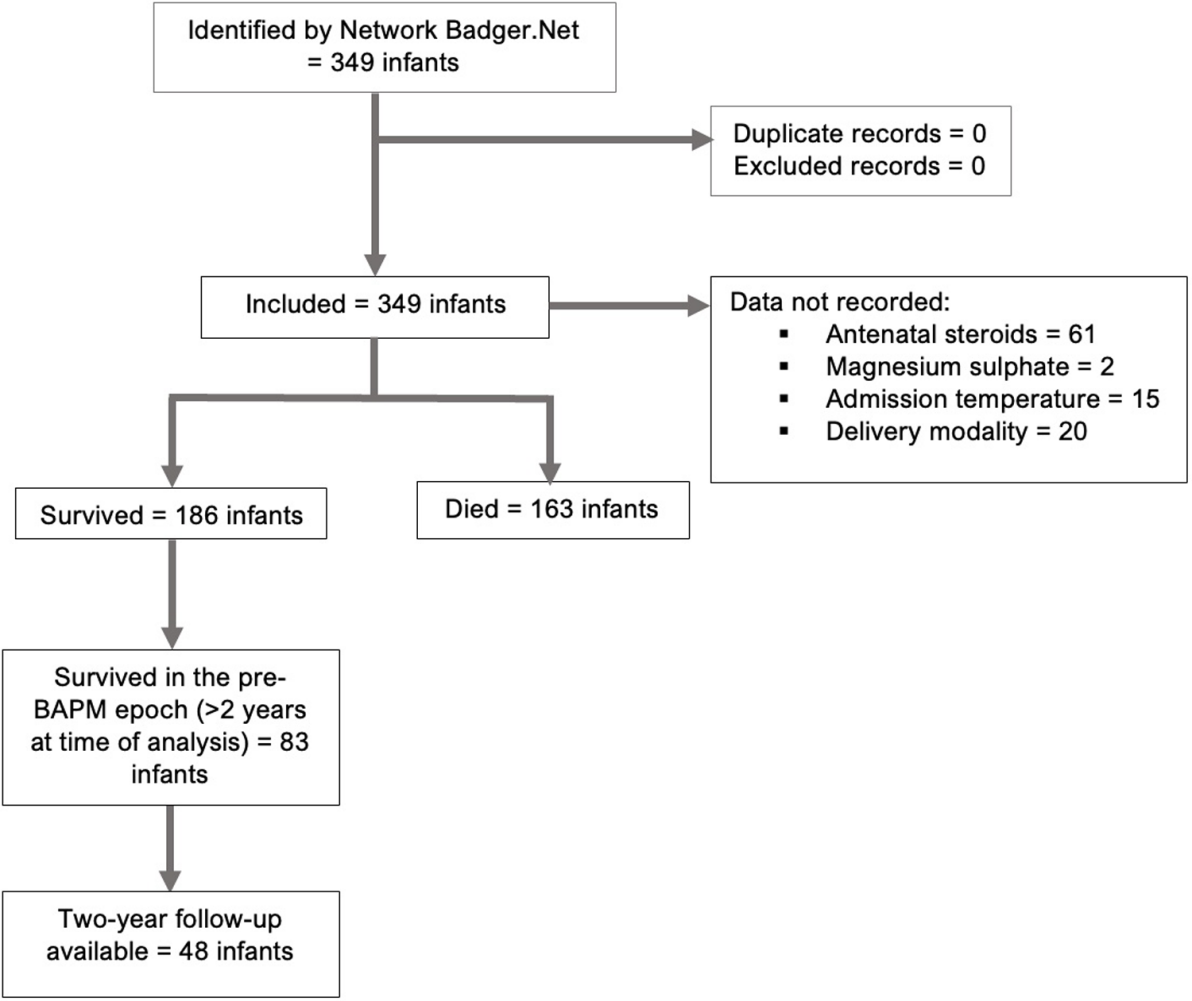
Consort diagram.

There were 349 extremely preterm infants (22 + 0–24 + 6 weeks) admitted to neonatal units across the NW network between 2018 and 2021 (Figure 1; Table 1). Focusing on periviable admissions (22 + 0–23 + 6 weeks) there were 160 admissions across the region, showing an unequal split with 25 infants delivering at 22 weeks and 135 delivered at 23 weeks. There were stable numbers of extremely preterm admissions pre- and post-BAPM, with 172 infants in the pre-BAPM cohort and 177 post-BAPM (Table 1). By gestational age categories, the 23-week cohort saw a significant decrease pre- and post-BAPM [76 infants pre- to 59 infants post-BAPM (22% decrease; *p* value <0.05)] whereas the 22-week cohort increased by 44% post-BAPM (from 9 to 16 infants). There was a non-significant 15% increase post-BAPM for 24-week infants (*p* value 0.19).

**Table 1:** Perinatal optimisation practices by pre- and post-BAPM epoch and associated infant mortality and morbidity rates.

		Pre-BAPM (2018–2019) *n*	%	Post-BAPM (2020–2021) *n*	%	*p*-Value
Number of births by gestation (weeks)	22 + 0–22 + 6	9	* *	16	* *	0.048*
	23 + 0–23 + 6	76	* *	59	* *	0.038*
	24 + 0–24 + 6	87	* *	102	* *	0.123
	Total	172	* *	177	* *	
Full antenatal steroids	22 + 0–22 + 6	3	*33*	8	*50*	0.676
	23 + 0–23 + 6	46	*61*	30	*51*	0.261
	24 + 0–24 + 6	63	*72*	71	*70*	0.672
	Total	112	*65*	109	*62*	
Any antenatal steroids	22 + 0–22 + 6	3	*33*	15	*94*	0.0029*
	23 + 0–23 + 6	63	*83*	50	*85*	0.773
	24 + 0–24 + 6	73	*84*	94	*92*	0.357
	Total	139	*81*	159	*90*	
MgSO4 provision	22 + 0–22 + 6	3	*33*	13	*81*	0.031*
	23 + 0–23 + 6	54	*71*	46	*78*	0.363
	24 + 0–24 + 6	74	*85*	88	*86*	0.812
	Total	131	*76*	147	*83*	
Delivery Method: Vaginal (including instrumental)	22 + 0–22 + 6	9	*100*	15	*94*	1
	23 + 0–23 + 6	62	*82*	48	*81*	0.974
	24 + 0–24 + 6	64	*74*	70	*69*	0.457
Delivery Method: CS (emergency or elective)	22 + 0–22 + 6	0	*0*	0	*0*	N/A
	23 + 0–23 + 6	5	*7*	8	*14*	0.241
	24 + 0–24 + 6	20	*23*	28	*27*	0.482
Survival	22 + 0–22 + 6	2	*22*	3	*19*	1
	23 + 0–23 + 6	26	*34*	23	*39*	0.567
	24 + 0–24 + 6	55	*63*	77	*75*	0.067
	Total	83	*48*	103	*58*	
Severe IVH (% of survivors)	22 + 0–22 + 6	0	*0*	0	*0*	N/A
	23 + 0–23 + 6	5	*19*	3	*13*	0.715
	24 + 0–24 + 6	7	*13*	12	*16*	0.689
	Total	12	*14*	15	*15*	
BPD (% of survivors)	22 + 0–22 + 6	2	*100*	3	*100*	1
	23 + 0–23 + 6	26	*100*	23	*100*	1
	24 + 0–24 + 6	52	*95*	74	*96*	0.693
	Total	80	*96*	100	*97*	

BAPM, British Association of Perinatal Medicine; MgSO4, magnesium sulphate; CS, caesarean section; IVH, intraventricular haemorrhage; BPD, bronchopulmonary dysplasia.

*Indicates significance <0.05.

*p*-value derived using *χ*^2^ (or Fisher's) test.

The italic values are the %, rather than raw figures.

### 3.1 Antenatal optimisation

Where there has been a decision for survival-focused care, then active antenatal care should be provided. The BAPM Framework emphasises key elements of antenatal optimisation, such as provision of antenatal steroids for lung maturation and improved morbidity and mortality, magnesium sulphate for neuroprotection and the importance of delivery in a centre with tertiary neonatal services to maximise outcome (1).

#### 3.1.1 Steroids

**Figure 2:**
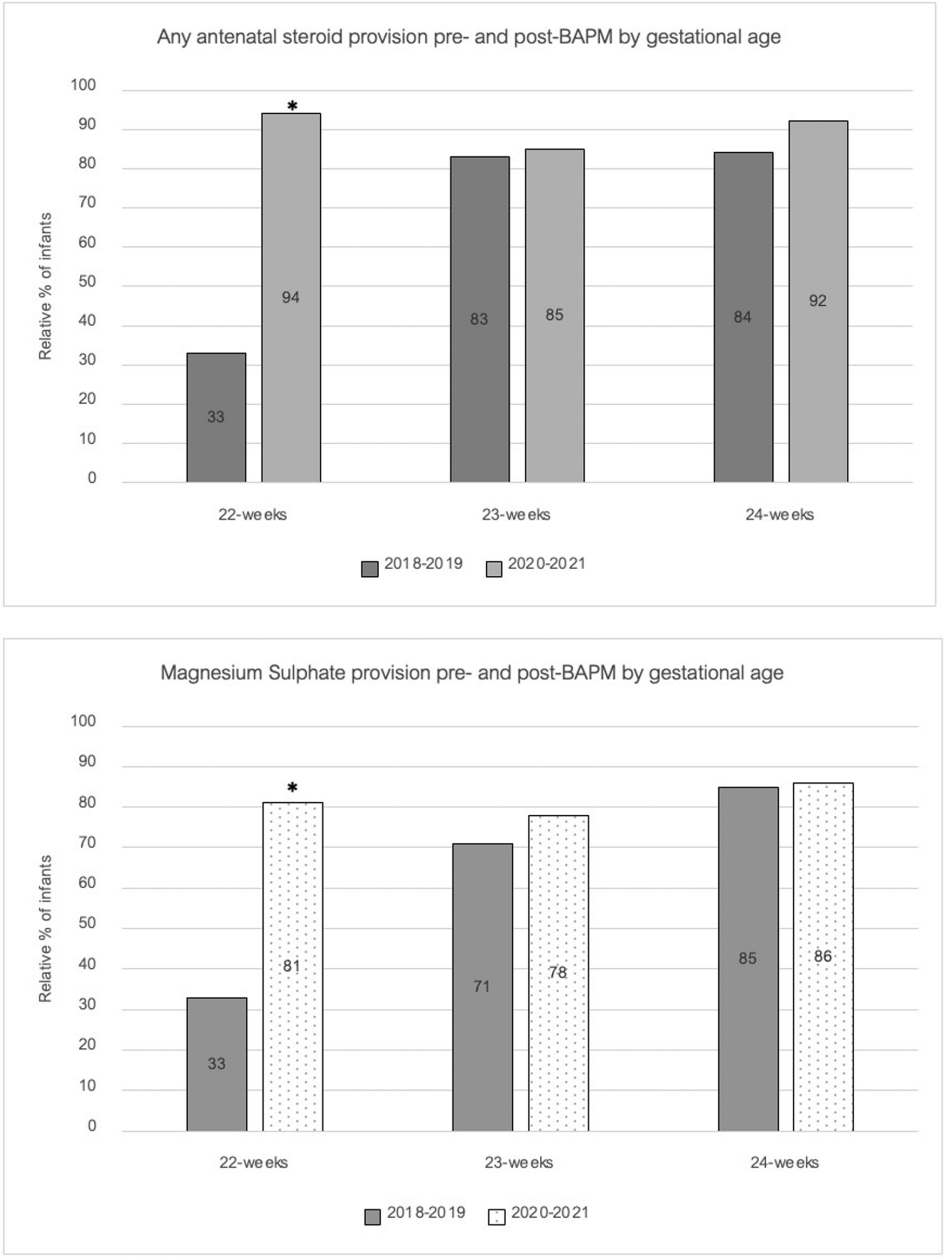
Antenatal optimisation.

Our data show differences in the rates of antenatal (AN) steroid administration across the gestational ages, with overall data showing 71% of 24-week infants received a full course of AN steroids compared to 56% of 23 week infants and 44% of 22-week infants. As Figure 2 shows, there was a marked impact pre- and post-BAPM on AN steroid administration, with the most pronounced effect in the 22-week cohort where provision of any doses of AN steroids increased from 33% to 94% (*p* value <0.01) and full AN steroids increased from 33% to 50% (*p* value = 0.67) (Table 1).

#### 3.1.2 Magnesium sulphate

A similar pattern was observed for magnesium sulphate (MgSO_4_^−^) administration to that seen for antenatal steroids. In the 24-week cohort magnesium sulphate was provided to the majority of mothers in labour (85%) and this remained static pre- and post-BAPM. Rates were lower in the 23-week cohort and demonstrated an insignificant rise post-BAPM from 71% to 78%. Conversely, for the 22-week cohort there was a significant increase in MgSO_4_^−^ post-BAPM with rates increasing from a mere 33% to 81% [*p* value = <0.05 (Figure 2)].

#### 3.1.3 Place of birth

There were high rates across the region for delivery of periviable infants in tertiary units which preceded the BAPM framework (Table 2). In the pre-BAPM epoch our data show 100% of babies born at 22 weeks (9 of 9) and 83% of babies born at 23 weeks (63 of 76) were delivered in a tertiary unit. These rates were unchanged post-BAPM with rates at 88% at 22 weeks (14 of 16) and 80% at 23 weeks (47 of 59).

**Table 2:** Periviable infant demographic data by epoch.

		22 + 0–22 + 6 weeks					23 + 0–23 + 6 weeks				
		Pre-BAPM (2018–2019)	%	Post-BAPM (2020–2021)	%	*p* Value	Pre-BAPM (2018–2019)	%	Post-BAPM (2020–2021)	%	*p* Value
Total infants		9	* *	16	* *		76		59		
	F	5	*56*	8	*50*	1.00	39	*51*	25	*42*	0.30
	M	4	*44*	8	*50*		37	*49*	34	*58*	
	Single	6	*67*	13	*81*	0.63	63	*83*	48	*81*	0.82
	Multiple	3	*33*	3	*19*		13	*17*	11	*19*	
Birth weight (grams)	Mean	517	* *	478	* *		581	* *	574		
	Min	400	* *	354	* *		388	* *	305	* *	
	Max	600	* *	689	* *		770	* *	750	* *	
	Q1	470	* *	465	* *		541	* *	520	* *	
	Q3	556	* *	542	* *		629	* *	633	* *	
	IQR	86	* *	77	* *		88	* *	113	* *	
	No >500 g	5	*56*	4	*44*	0.20	66	*87*	51	*86*	0.95
Antenatal steroids			* *		* *			* *		* *	
	Full	3	*33*	8	*50*	0.68	46	*61*	30	*51*	0.26
	Incomplete	0	*0*	7	*44*	1.00	17	*22*	20	*34*	0.14
	None	0	*0*	0	*0*		0	*0*	1	*2*	
	Unknown	6	*67*	1	*6*		13	*17*	8	*14*	
Magnesium sulphate			* *		* *			* *		* *	
	Yes	3	*33*	13	*81*	0.03*	54	*71*	46	*78*	0.36
	No	6	*67*	3	*19*		22	*29*	13	*22*	
Admission temperature (celcius)			* *		* *			* *	* *		
	Mean	36.3	* *	36.6	* *		36.5	* *	36.6		
	Min	34.6	* *	35.5	* *		33.4	* *	34.0		
	Max	37.1	* *	37.7	* *		39.5	* *	38.7		
	Q1	36.0	* *	36.2	* *		36.1	* *	36.3		
	Q3	36.9	* *	37.1	* *		37.1	* *	37.0		
	IQR	0.9	* *	0.9	* *		1.0	* *	0.7	* *	
Outcome			* *		* *			* *	* *	* *	
	Survived	2	*22*	3	*19*	1.00	26	*34*	23	*39*	0.57
	Died	7	*78*	13	*81*		50	*66*	36	*61*	
Location of delivery	NICU	9	*100*	14	*88*	0.52	63	*83*	47	*80*	0.63
	LNU	0	*0*	2	*13*		13	*17*	11	*19*	
	SCBU	0	*0*	0	*0*		0	*0*	1	*1*	
Timing of death (where applicable)	First 24 h	4	*57*	5	*38*		12	*24*	7	*19*	
	First 72 h	0	*0*	2	*15*		6	*12*	2	*6*	
	First 14 days	2	*29*	4	*31*		17	*34*	12	*33*	
	First 30 days	0	*0*	0	*0*		8	*16*	5	*14*	
	>30 days	1	*14*	2	*15*		7	*14*	10	*28*	
	Total	7	*100*	13	*100*		50	100	36	*100*	

BAPM, British Association of Perinatal Medicine; F, female; M, male; Q1, Lower Quartile; Q3, Upper Quartile; IQR, interquartile range; NICU, neonatal intensive care unit; LNU, local neonatal unit; SCBU, special care baby unit.

*Indicates significance <0.05.

*p*-value derived using *χ*^2^ (or Fisher's) test.

The italic values are the %, rather than raw figures.

### 3.2 Outcomes

#### 3.2.1 Survival

The study data show increasing survival rates with increasing gestational age; 20% survival for the 22-week cohort, 36% for 23-week cohort and 70% for 24-week cohort (Table 1). For surviving infants, the majority had a diagnosis of BPD [343 of 349 infants (98%)] with only 6 infants (2%) not receiving a diagnosis of BPD at discharge (Table 1). These 6 infants without BPD were all >24 weeks with a mean birth weight 668 grams (IQR: 636–675 grams). Five of these six infants had received a full course of antenatal steroids (the remaining infant did not have their AN steroid status recorded).

There were 27 surviving infants who experienced a severe intraventricular haemorrhage (Grade 3 or 4) (Table 1). There were no severe IVH's in the surviving 22-week cohort. Severe IVH did occur in the 23- and 24-week surviving infants (8 and 19 infants respectively). There was an equal distribution of severe IVH across the sexes (13 female infants and 14 male infants). Median birth weight was 659 grams (IQR: 583–729 grams). The majority of infants with severe IVH had been born in a tertiary unit (74%) and had received magnesium sulphate (81%). In 59% of cases the infant had received a full course of AN steroids, rising to 85% of these infants receiving at least one dose of AN steroids. Provision of a complete course of antenatal steroids was associated with decreased odds of developing a severe IVH [OR: 0.43 (95% CI: 0.2–1.0; *p* value 0.05); Table 3].

**Table 3:** Odds ratios for significant periviable and extremely preterm survivor outcomes.

Periviable survival rates pre- and post-BAPM			
	Pre-BAPM	Post-BAPM	
Survived	28	26	
Died	57	49	
	Odds ratio	95% confidence interval	*p* value
	1.08	0.56–2.08	0.81
Risk of death following complete antenatal steroid provision			
Gestational age (weeks)	Odds ratio	95% confidence interval	*p* value
22 + 0–22 + 6	0.13	0.01–1.45	0.098
23 + 0–23 + 6	0.31	0.15–0.67	0.003
24 + 0–24 + 6	0.67	0.34–1.3	0.235
Risk of IVH in extremely preterm survivors following complete antenatal steroid provision			
	Severe IVH	Non-severe IVH	
Complete antenatal steroids	16	123	
Nil/incomplete antenatal steroids	11	36	
	Odds ratio	95% confidence interval	*p* value
	0.43	0.2–1.0	0.05

#### 3.2.2 Periviable survivors

The data show modest survival rates at periviable gestations. In the 22-week cohort the survival rate was 20% (5 of the 25 infants). Survival increased in the 23-week cohort to 36% (49 from 135 infants). The average birth weight was higher in the surviving cohort; 503 g (survivors) vs. 489 g (deceased) for 22 weeks and 587 g (survivors) vs. 574 g (deceased) for 23 weeks. Of note, the smallest periviable birth weights occurred in survivors (lowest weight 354 grams for 22-weeks and 305 grams for 23-weeks). Comparison of periviable survival rates pre- and post-BAPM shows no significant difference between the two epochs [OR 1.08 (95% CI = 0.56–2.08; *p* value 0.81); Table 3].

Surviving infants had significantly increased rates of AN steroid exposure compared to infants that died. At 22-weeks 80% (4 out of 5) of survivors had received a full course of antenatal steroids, compared to a mere 35% of 22-week infants that died (*p* value <0.01). For 23-week infants, 73% of survivors had received full antenatal steroids compared to 36% of those that died (*p* value <0.01). This data is observational only and therefore cannot comment on causation. The data do show an association between increased antenatal steroid administration and increased odds of survival (Table 3).

All periviable (22 + 0–23 + 6 weeks) survivors had a diagnosis of bronchopulmonary dysplasia. Grade 3 or 4 IVH occurred in eight of the surviving 23-week infants (16%) and there were no cases in surviving 22-week infants.

#### 3.2.3 Infants that died

For those periviable infants who did not survive, we examined the timing of death. In the 22-week cohort the data show that the majority (55%) died within the first 72 h of life and 75% died within the first 10 days of life. For the 23-week cohort the timing of death profile shows a more gradual distribution over the first weeks of life (23% in the first 72 h, 45% in the first 10 days and 64% within the first month of life).

### 3.3 Periviable transfers

**Figure 3:**
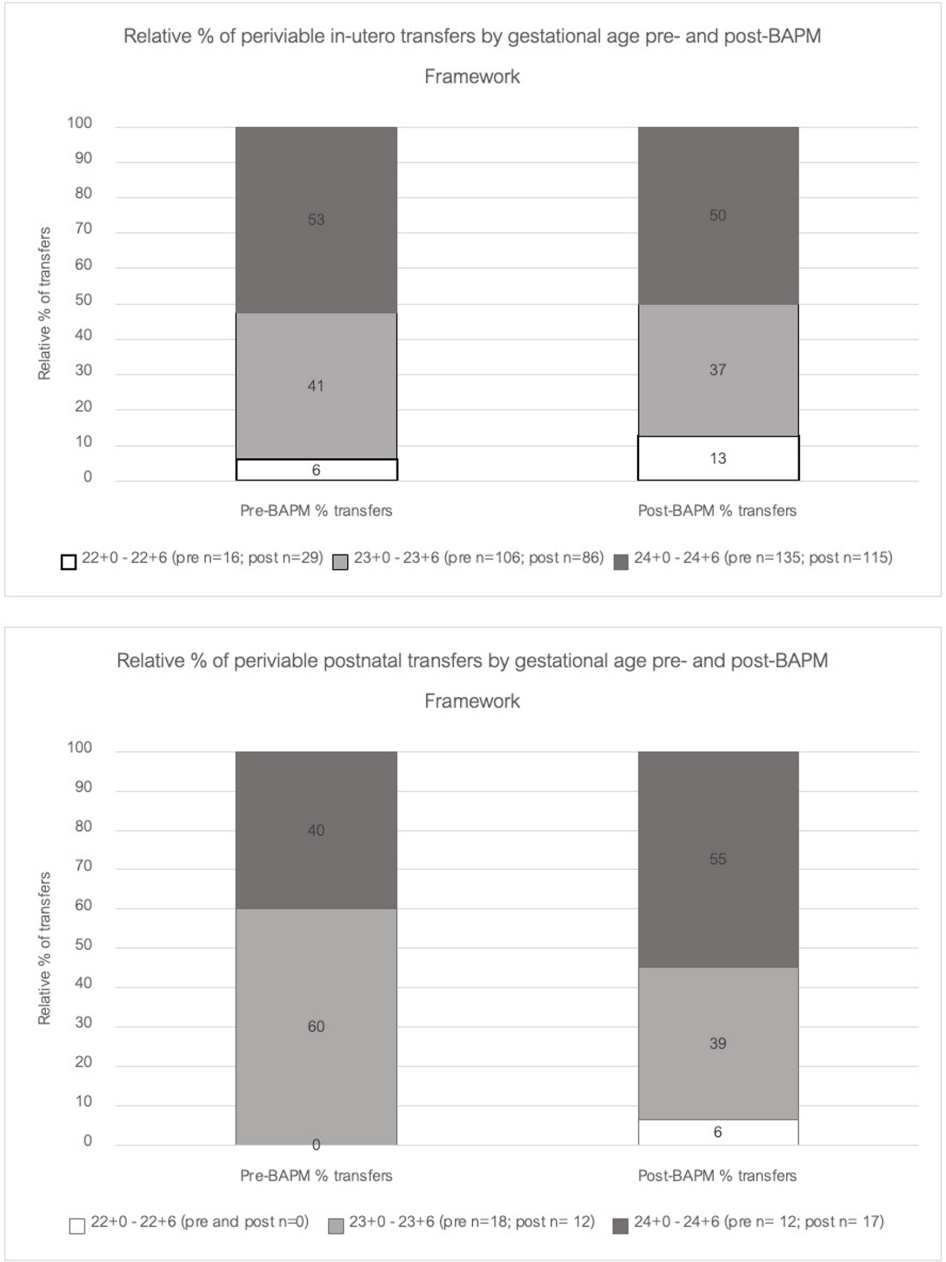
Transfer rates by gestation pre- and post-BAPM.

An additional aim of this study was to evaluate the impact of the BAPM framework on periviable transfers across the region. There was an overall decrease of 10% in total periviable transfers between the pre- and post-BAPM epochs (Figure 3). This decrease was seen in the 23 + and 24 + week cohorts. Despite the overall decrease in periviable transfers in the post-BAPM epoch, data gathered from the ConnectNW transfer records confirm that there has been a statistically significant increase for in-utero transfers of 22 + 0–22 + 6 week infants after the introduction of the BAPM framework (*p* value = 0.03). The relative percentage of 22-week in-utero transfers within the network rose from 6% pre-BAPM (16 mothers) to 13% post-BAPM (29 mothers). Relative percentage of in-utero transfers for 23- and 24-week threatened deliveries remained static with no significant differences pre- or post-BAPM.

## 4 Discussion

This study aimed to evaluate the clinical impact of the revised BAPM framework for periviable infants across the North West of England. The data shows that since the introduction of the revised BAPM framework increased numbers of 22-week and 23-week infants are receiving survival focused care. The data show an improvement in rates of antenatal optimisation (antenatal steroids, magnesium sulphate provision) in the post-BAPM epoch, particularly in the 22-week cohort. This suggests the BAPM framework has impacted practice by promoting a more concordant approach of aligning antenatal optimisation with the decision for postnatal survival-focused care. Whilst this is a logical improvement in approach–if we are considering intensive care, then the infant should receive antenatal optimisation–there remain discrepancies in rates of optimisation depending on gestational age. This is particularly evident in the administration of antenatal steroids. Despite an improvement in administration of any doses of antenatal steroid from 33% to 94% in the post-BAPM epoch for 22-week infants, when we look specifically at administration of a complete course of antenatal steroids for 22-week infants this improvement drops to 50% (Table 1). Conversely, for infants delivered in the 24-week cohort, administration of a complete course of AN steroids was 72% and 70% pre/post-BAPM (Table 1). This finding requires further evaluation to identify targets for improvement in steroid provision to the earlier gestation infants who are receiving active-survival focused care. The lower rates of AN steroids in the 22- week cohort may represent the time delay intrinsic in the practicalities of conducting pre-birth decision-making conversations between clinicians and parents at 22- and 23-weeks. There can be delays between parents presenting in suspected labour at 22-weeks and the senior obstetric and neonatal clinicians being available to have detailed discussions with parents about management at birth and appropriateness of antenatal optimisation. Data from other centres clearly demonstrates an association between antenatal steroid administration and survival in 22-week infants, with provision of AN steroids doubling the likelihood of survival compared to postnatal survival-focused care alone (14).

**Figure 4:**
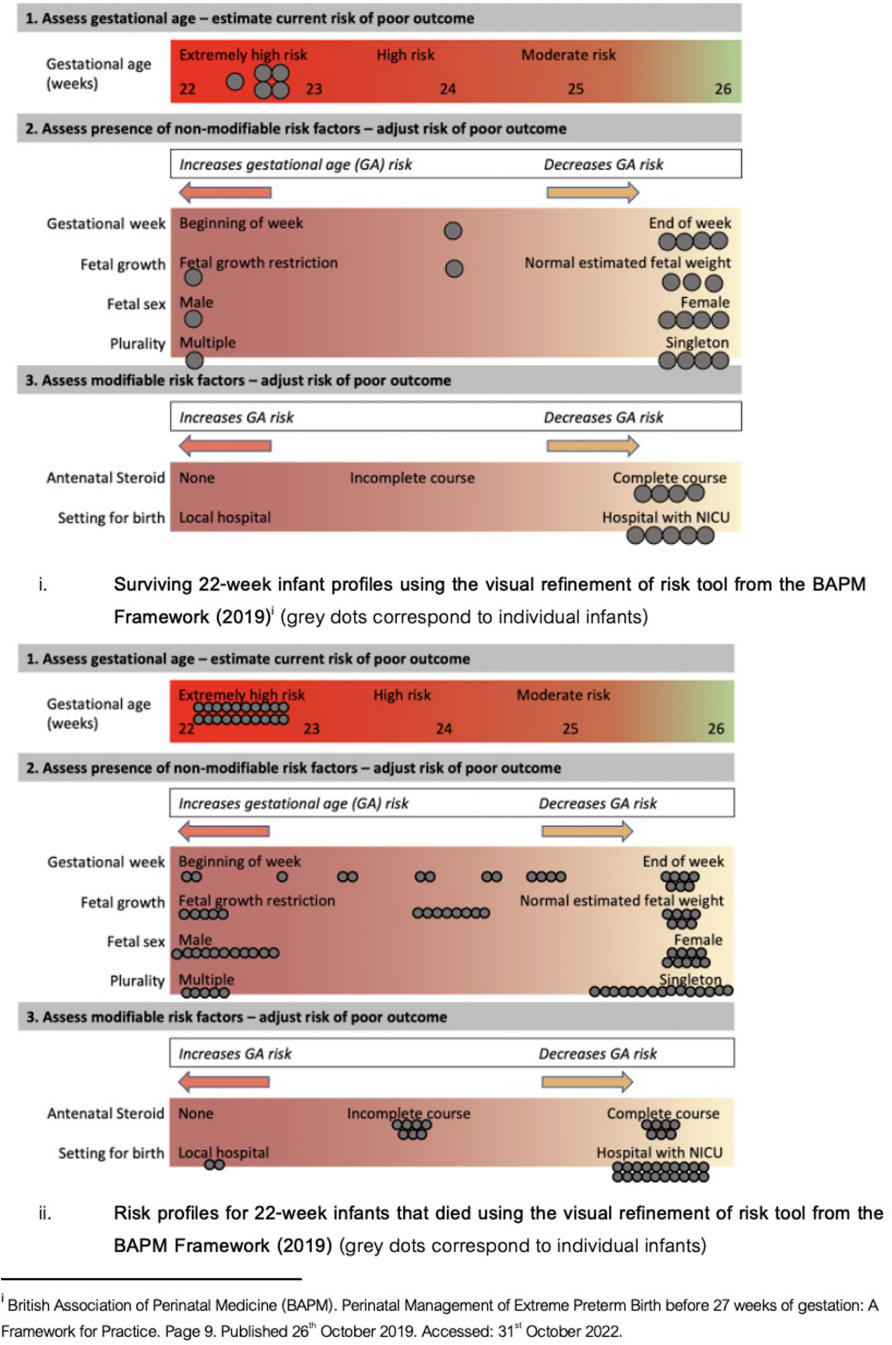
BAPM risk profiles.

Our data show a minimal increase in complete AN steroid provision from 33% to 50% pre-/post-BAPM. This may contribute to the static survival rates in this gestational age cohort between the two epochs (survival at 22-weeks remained 22% pre- and 19% post-BAPM). All surviving 22-week infants received a complete course of antenatal steroids (one survivor had missing data for steroid administration). Utilising the risk refinement tool from the BAPM framework [page 9 (1)], each of the 22-week surviving infants would have been deemed extremely high/high risk (Figure 4). All infants born at 22 + 0–22 + 2 weeks died. Within the subgroup of 22-week infants that died there was a spectrum of protective and detrimental risk factors for poor outcome (Figure 4). Within the 22-week cohort, there were 10 infants who died that had numerous protective factors (born at the end of the week, singleton, appropriate growth, complete antenatal steroids) and therefore, had similar pre-delivery risk profiles as those 22 week infants who survived. Whilst risk profiles do not claim to guarantee a particular outcome, our data support how non-specific these clinical risk factors are when applied to an individual infant and their outcome.

### 4.1 Selection of outcome statistics in pre-delivery discussions

Given the limited predictive value of the pre-birth risk profiles when applied to individual infants, it is difficult for clinicians to be able to determine with any accuracy which infants will survive, and which will not. This makes pre-birth discussions with the parents complex. Sincere and authentic discussions between the clinician and parent are needed, to establish, in the face of significant uncertainty, whether or not they feel pursuing intensive, survival-focused care is in their infant and their family's interest.

The use of predictive statistics in this type of pre-delivery discussion is fraught with difficulty (15, 16). Our data show variation from the survival rates cited in the BAPM framework (1). Survival rates for 24-week infants were improved over the BAPM figures (70% regional survival vs. 60% quoted by BAPM). At 23 weeks BAPM quote a comparable 40% survival rate to our regional rate at 36%. However, in the 22-week cohort our survival rate was 20%, in comparison to the BAPM 30% survival rate. This is a clinically relevant difference and may well have an impact on both the clinician and parental decision-making process. Whilst statistics do not apply to the individual, they do provide valuable information in guiding likelihood decision-making. The issue of which statistics should be used, where requested by parents and to inform clinician decision-making, is problematic. Clinicians may not have access to their local unit data, which may have such infrequent periviable cases that local data is rendered uninterpretable. However, presentation of regional or national data with mixing of outcomes from level 1, 2 and 3 units presents a false image [research has shown outcomes improve if these infants are delivered in a tertiary unit (17)] and presenting inaccurate or incomplete information to parents has the potential to create moral distress dilemmas for the clinician involved in the conversation (18).

### 4.2 Timing of death

**Figure 5:**
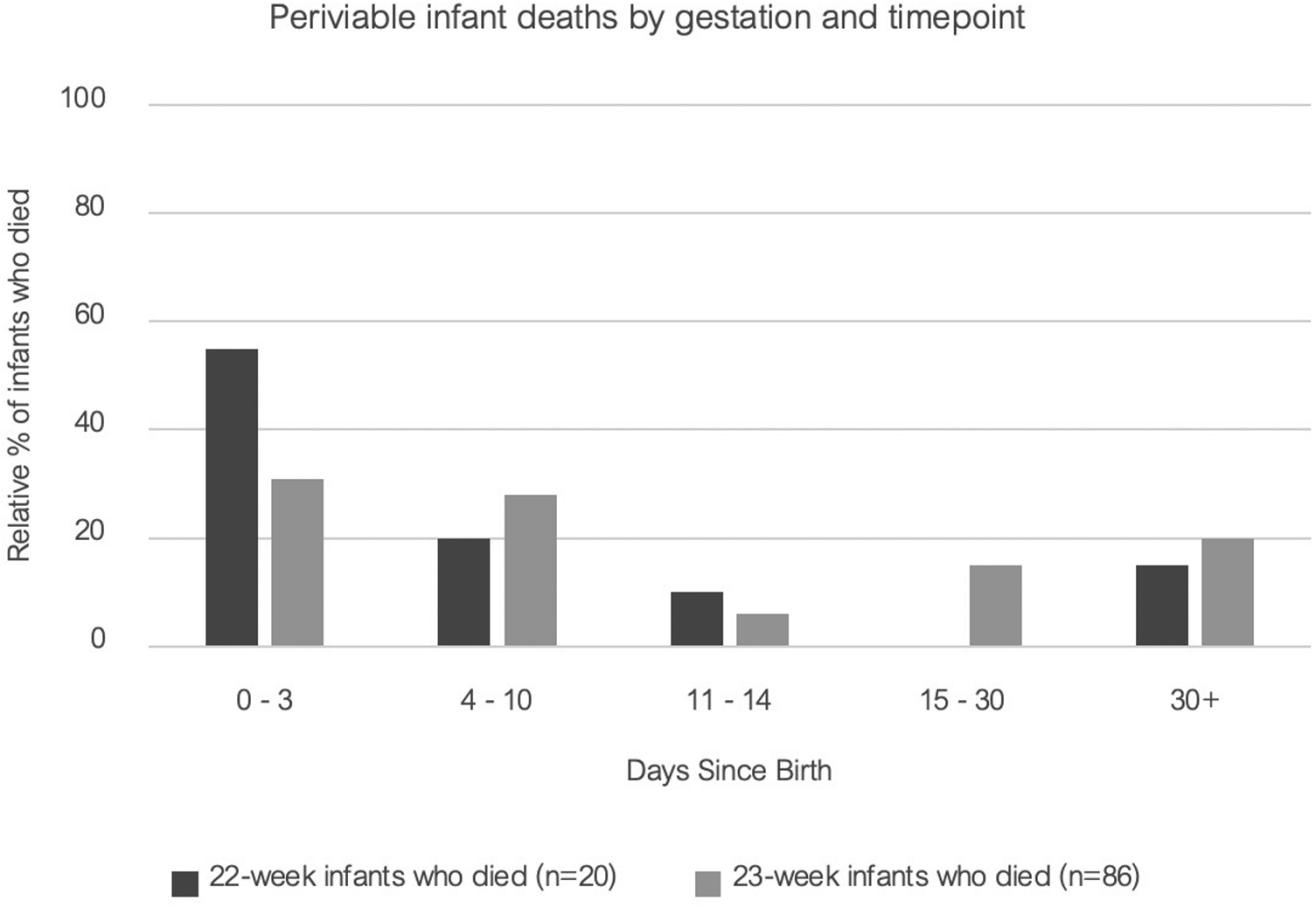
Periviable infant deaths by gestation and timepoint.

Our data also demonstrate that for 22-week infants who did not survive, the majority (55%) died within the first 72 h of life, and 75% died within the first 10 days of life (Figure 5). This would indicate that where a trial of life has been attempted the likelihood of survival was clarified early in the admission. One of the central tenets of medical ethics is to “Do No Harm” (19). Application of this guiding principle can be problematic when attempting to determine pre-delivery the most appropriate course of action in relation to periviable infants. Intensive care is an invasive, brutal environment and clinicians may carry a burden of guilt for putting infants through this where they have poor odds of survival (20). Our data demonstrate that in cases where a trial of life has been attempted, it may become apparent early in the admission whether intensive care will be unsuccessful for these infants. A trial of life in these circumstances can be appropriate to ensure that infants with protective risk factors (as outlined in the BAPM framework) receive a chance at survival, and in cases where it becomes clear this is not working, their families have been given precious time with their baby before they die. Parallel planning and robust palliative care practices are essential for periviable infants and their family. As outlined by Fawke et al, palliative and end of life care is an active process and should be managed with “the same degree of skill as if the baby was to receive intensive care” (21). Senior medical and nursing staff are essential throughout this process to balance having the requisite skills to provide intensive care to these infants and the experience to recognise when this is not working and, in collaboration with the parents, transition to end of life care where appropriate.

### 4.3 Impact on transport services

An additional aim of this study was to evaluate impact of the revised BAPM framework on the regional transport service. Data from this study showed an overall decrease in total periviable transfers between the pre- and post-BAPM epochs (Figure 3). This may be due to the impact of the COVID-19 pandemic. The consequent periods of national lockdown, particularly in 2020, were associated with decreased presentations to hospital (22). The ConnectNW data indicate reduced in-utero transfers at 23- and 24-weeks gestation in the 2020–2021 epoch which may reflect reduced rates of threatened preterm labour in these cohorts, or reluctance to transfer antenatally for infection control reasons during the pandemic. Postnatal transfer rates were static pre- and post-BAPM (30 and 31 infants respectively) indicating that if there had been reluctance to transfer antenatally during the pandemic this did not translate into increased liveborn deliveries requiring subsequent postnatal transfers. Our regional dataset shows stable numbers of extremely preterm infants being born and admitted to NICU across the pandemic (Table 1) and notes no significant difference in place of birth pre- and post-BAPM (Table 2).

### 4.4 Limitations

Inherently, data gathered from Badger.Net will only report for infants where survival-focused care was attempted at delivery and the infant was admitted to NICU. The dataset does not reflect approaches to infants where there had been an antenatal decision for comfort-focused delivery room care, or cases where the infant did not respond to interventions and died in delivery suite. The data instead reflect infants where survival-focused care was deemed appropriate by the parent-clinician team. In these cases, one would anticipate that the infant should have received maximal perinatal optimisation. If the decision is for active management at birth, the infant should be given the best chance of this being successful with provision of antenatal optimisation, such as antenatal steroids and magnesium sulphate. By analysing data from Badger.Net (only infants admitted to NICU), our dataset evaluates for discordance between the decision for survival-focused care at delivery and provision of antenatal optimisation measures. Therefore, our dataset was suitable for the aims of this study.

The data extraction process was unable to download accurate information relating to retinopathy of prematurity screening results. This is due to variation between units about where ROP screening information is entered into Badger.Net. Therefore, whilst the initial plan was to include ROP as one of the short-term outcome measures, the available data did not allow this. Therefore, ROP grading was not included in this study.

The research team had wanted to examine the 2-year follow-up outcomes but this was not possible due to incomplete data. This was focused on the pre-BAPM epoch (2018–2019) as these infants were all >2 years when the data extraction was performed. Data show suboptimal rates of recorded 2-year follow-up with only 48 of 83 surviving infants (58%) having a recorded follow-up on Badger.Net.

## 5 Summary

The launch of the revised BAPM Framework in late 2019 has impacted periviable perinatal optimisation practices in the North West region. Our data demonstrate improvements in perinatal optimisation; however, there continues to exist significant variation between optimisation rates between the gestational ages, with reduced perinatal optimisation in the youngest gestational age cohort, even where survival-focussed care is being implemented after birth. The proportion of survivors remains static between the pre- and post-BAPM epochs and the underlying reasons for this remain to be ascertained. Our data, albeit limited by total number of 22-week infants, show an association between perinatal optimisation and survival, with all surviving 22-week infants receiving a complete course of antenatal steroids and delivered within a tertiary neonatal unit.

Given our current inability to accurately predict the ultimate outcome for infants born at periviability, clinician conversations with parents must centre on the individual circumstances and parental perspective. We need to provide as accurate a representation as possible of the various protective and detrimental factors as relate to that individual infant, whilst also discussing the perspective of the parents in balancing the risks and benefits in relation to the value of life, death, disability and the harm intrinsic to intensive care. These discussions are intensely personal, wide-ranging, require time and sensitivity to be able to delve and explore, rather than dictate, the options with the parents. Current practices centre on information being provided by the clinician once parents present in threatened periviable labour. This is problematic due to the high emotional demands on the parents during this time and the potential time-constraints depending on how rapidly the labour progresses. Further study is required to optimise information sharing with parents facing periviable labour.

## Data Availability

The anonymised data supporting the conclusions of this article will be made available by the corresponding author, without undue reservation.
